# Registry-Based Assessment of Shunt Operation Methods and Outcomes in Idiopathic Normal Pressure Hydrocephalus (RASHOMON Study): Protocol for a Multicenter Prospective Observational Cohort Study

**DOI:** 10.2196/80678

**Published:** 2025-11-21

**Authors:** Eriko Okumura, Kazuhito Takeuchi, Hiroyuki Momota, Tetsuya Nagatani, Toshiaki Taoka, Yuri Aimi, Atsushi Hashizume, Masaki Okazaki, Ryuta Saito

**Affiliations:** 1 Department of Neurosurgery Graduate School of Medicine Nagoya University Nagoya Japan; 2 Department of Neurosurgery National Center for Geriatrics and Gerontology Obu Japan; 3 Department of Neurosurgery Japanese Red Cross Aichi Medical Center Nagoya Daini Hospital Nagoya Japan; 4 Department of Innovative Biomedical Visualization (iBMV) Graduate School of Medicine Nagoya University Nagoya, null Japan; 5 Department of Advanced Medicine, Department of Clinical Research Education Nagoya University Hospital, Graduate School of Medicine Nagoya University Nagoya Japan; 6 Department of Nephrology Graduate School of Medicine Nagoya University Nagoya Japan

**Keywords:** idiopathic normal pressure hydrocephalus, ventriculoperitoneal shunt, lumboperitoneal shunt, ventriculoatrial shunt, observational study, registry, cerebrospinal fluid, outcomes, Japan, protocol

## Abstract

**Background:**

Idiopathic normal pressure hydrocephalus is an age-related condition characterized by cerebrospinal fluid accumulation and ventricular enlargement, leading to cognitive decline, gait disturbance, and urinary incontinence. Although shunt surgery is the primary treatment, the optimal surgical strategy remains uncertain, and procedure selection is often not tailored to individual patient characteristics. Notably, no prospective study has directly compared the 3 major shunt techniques (ie, ventriculoperitoneal, lumboperitoneal, and ventriculoatrial).

**Objective:**

This multicenter prospective observational study will aim to generate high-quality clinical evidence by evaluating the effectiveness and safety of the 3 aforementioned surgical options in a real-world setting.

**Methods:**

Patients suspected of having this condition will be enrolled based on characteristic symptoms and imaging findings, with a spinal tap test recommended but not mandatory. Eligible patients will undergo one of the aforementioned surgical procedures.

**Results:**

On the basis of recent data from 11 collaborating institutions in Japan, we estimate enrolling 278 cases: 188 (67.6%) surgical, 100 (36%) nonsurgical, and 10 (3.6%) dropouts. Clinical outcomes will be assessed at baseline; after the tap test (if performed); and at 1 week, 3 months, and 12 months postoperatively. The analyses will explore the associations between outcomes and surgical methods, patient backgrounds, and imaging features. As of May 2025, approximately 60 participants have been enrolled from 11 institutions. Data collection is ongoing and is expected to be completed by December 2026. There will be a 1-year follow-up period. The main study results are anticipated to be published in 2028.

**Conclusions:**

Japan is well placed to lead this comparative study with its extensive experience in diagnosing and treating this disease. These findings are expected to provide practical guidance for individualized surgical decision-making and contribute to the global consensus on optimal treatment strategies.

**Trial Registration:**

Japan Registry of Clinical Trials jRCT1040250005; https://jrct.mhlw.go.jp/latest-detail/jRCT1040250005

**International Registered Report Identifier (IRRID):**

DERR1-10.2196/80678

## Introduction

One disease closely related to aging is idiopathic normal pressure hydrocephalus (iNPH) in an aging society. The cause of iNPH is believed to be a disorder of cerebrospinal fluid (CSF) dynamics [1,2], leading to the accumulation of CSF within the cranial cavity and resulting in characteristic brain morphological changes, including ventricular enlargement. The Primary clinical symptoms include cognitive dysfunction, gait disturbance, and urinary incontinence [2-4]. This condition significantly impairs physical and mental function over several years, even up to decades after its onset, causing disruptions in daily life and shortening life expectancy. One study found that the 5-year mortality rate in untreated older patients with iNPH was significantly higher (87.5%) than in an age-matched healthy population (19.1%) [5]. Individuals aged 60 years and older are at risk of developing iNPH. A Japanese epidemiological survey reported that the population aged ≥65 years is approximately 36.2 million, accounting for 29.3% of the total population in 2024 [6]. In addition, the prevalence of iNPH ranges from 0.37% to 2.9% [7-9]. An estimated 12,900 new patients with iNPH visit health care facilities annually in Japan [10], suggesting that the number of potential patients is even higher.

The world’s first guidelines for the management of iNPH were published in Japan in 2004, and several advancements have since been made in the diagnosis and treatment of this condition [11]. The characteristic brain imaging findings are disproportionately enlarged subarachnoid space hydrocephalus (DESH) [11-13]. The tap test, a CSF drainage test, is an important diagnostic tool. These findings also allow for the prediction of treatment efficacy [11,14,15]. Generally, the treatment preferred is shunting, using 1 of the 3 surgical methods: ventriculoperitoneal shunt (VPS), lumboperitoneal shunt (LPS), or ventriculoatrial shunt (VAS). In 2010, the results of the prospective clinical study, Study of Idiopathic Normal Pressure Hydrocephalus on Neurological Improvement (SINPHONI), which investigated the efficacy of VPS for iNPH, were published, demonstrating the effectiveness of VPS [13]. In 2015, the results of the SINPHONI-2 trial, which evaluated the efficacy of LPS for iNPH, showed that LPS surgery yielded results comparable to VPS [16]. However, no prospective studies have reported on the efficacy of VAS for iNPH. Although the third edition of the Japanese iNPH clinical practice guidelines was published in 2020, it does not compare the 3 surgical methods or provide recommendations on which one to choose [17]. Thus, the choice remains at the discretion of individual institutions and surgeons based on their experience and clinical judgment.

This study aims to evaluate and compare the effectiveness and adverse events associated with 3 surgical approaches for iNPH: VPS, LPS, and VAS. It will aim to provide evidence-based guidance for selecting the most appropriate surgical method for iNPH treatment.

## Methods

### Study Design

This multicenter prospective cohort observational study will be conducted from November 2024 to December 2028. The patient enrollment period is planned to run for approximately 2 years, with a follow-up period of 1 year. This study will evaluate and compare the effectiveness and adverse events associated with 3 surgical approaches for iNPH: VPS, LPS, and VAS. The goal is to provide evidence-based guidance for selecting the most appropriate surgical method for iNPH treatment.

### Study Settings

This study plans to register patients from 11 institutions, including Nagoya University Hospital and its collaborating institutions. These institutions were selected through a fair process based on their agreement with the objectives of this study and their capability to provide comprehensive care for patients with iNPH. The iNPH database committee was organized by recruiting core members, including 5 neurosurgeons and 1 neuroradiologist, from Nagoya University Hospital and its affiliated institutions. The committee developed clinical trial plans and will manage and centrally review the data as independently as possible. Specifically, 1 neuroradiologist and 2 neurosurgeons will judge the anonymized images and the observed data of patients who are not involved in their treatment. Timely reminders will be issued to the attending physicians to minimize the risk of missing follow-up data.

### Study Population and Eligibility Criteria

This study will include patients who are suspected of having iNPH based on head imaging and clinical symptoms and who undergo a lumbar puncture tap test. Among these patients, those deemed to require surgical treatment for hydrocephalus will be offered the option to choose 1 of the following procedures: VPS, LPS, or VAS. The diagnostic and treatment algorithm generally follows the third edition of the Japanese iNPH clinical practice guidelines. However, based on previous clinical experience, a small number of patients with an Evans index of less than 0.3 may still exhibit features suggestive of iNPH. To allow for the inclusion of such cases, the callosal angle will be incorporated as an additional imaging criterion. Moreover, the criterion of “normal CSF protein levels” described in the guidelines is not a mandatory requirement in this study; values less than or equal to 100 mg/dL are considered acceptable. The inclusion and exclusion criteria are presented in Textbox 1.

**Inclusion and exclusion criteria.**

**Inclusion criteria**

- Aged 60 to 85 years
- Having at least 1 of the following symptoms: gait disturbance, cognitive impairment, or urinary incontinence
- Presents with ventricular enlargement (Evans index >0.3) or a small callosal angle (<90°)
- Head magnetic resonance imaging or computed tomography showing—DESH (disproportionately enlarged subarachnoid-space hydrocephalus) findings, including narrowing of the subarachnoid space in the high-convexity and midline regions

**Exclusion criteria**

- Informed consent cannot be obtained
- Presence of a clearly identifiable underlying condition known to cause hydrocephalus
- A clearly identifiable space-occupying lesion in the cranium
- Significant brain atrophy observed due to stroke or trauma.
- A history of intracranial surgery for neurological disorders, excluding minor procedures (eg, evacuation of chronic subdural hematoma).
- The presence of systemic diseases (such as heart failure, respiratory failure, liver failure, or kidney failure) or cancer that makes the participant unsuitable
- The participant deemed unsuitable by the principal investigator or study collaborators

### Sample Size Calculation

**Figure 1:**
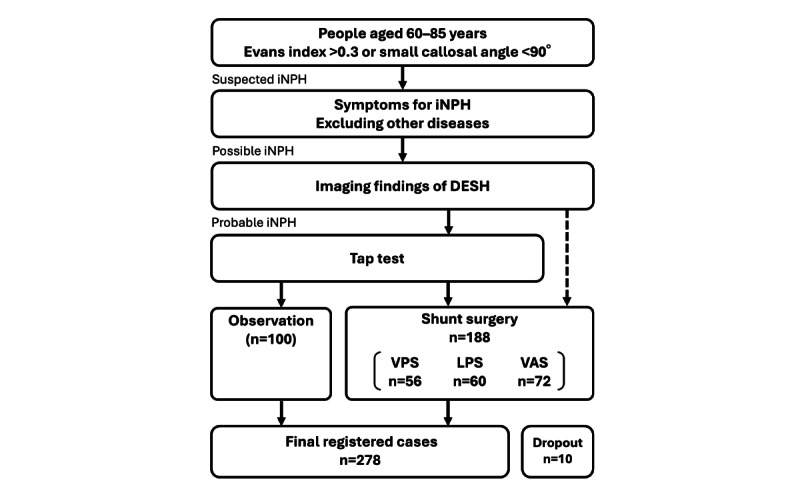
Flowchart of the patient enrollment criteria and overall study design in this multicenter prospective observational study of idiopathic normal pressure hydrocephalus (iNPH). Observation cases are determined based on the patient’s intention, condition, and need for surgery. Shunt procedures (ie, ventriculoperitoneal shunt [VPS], lumboperitoneal shunt [LPS], or ventriculoatrial shunt [VAS]) are selected according to the patient’s clinical status and surgeon’s preference. The target numbers for the shunt procedures were estimated from the number of previous surgical cases. DESH: disproportionately enlarged subarachnoid space hydrocephalus.

The sample size was calculated based on reports of previous iNPH shunt procedures. On the basis of the iNPH cases at the collaborating research institutions over the past 2 years, 56 (29.8%) VPS, 60 (31.9%) LPS, and 72 (38.3%) VAS procedures are anticipated to be performed, resulting in a total of 188 surgical cases during the 2-year enrollment period. In addition, 100 nonsurgical cases are expected to be included during the same period. Considering a 5% dropout rate during follow-up among the operated cases, the final sample size is estimated to be 278 cases (Figure 1).

### Outcome Measures

The primary outcome is the change in the Modified Rankin Scale (mRS) score from baseline to 1 year after surgery in the VAS group compared to the VPS+LPS group.

The secondary outcomes are mentioned subsequently. First, the change in the mRS score from baseline to 1 year after surgery in the VAS group will be compared with that in the VPS group. Second, the change in the mRS score from baseline to 1 year after surgery in the VAS group will be compared with that in the LPS group. Third, the change in the mRS score from baseline to 3 months after surgery in the VAS group will be compared with the VPS+LPS group. Fourth, the occurrence of adverse events at 3 months and 1 year after surgery in the VAS group will be compared with the VPS+LPS group. Fifth, the evaluation items influencing treatment decisions in surgical versus nonsurgical cases will be compared.

### Data Collection

The attending physicians and supporting medical staff will document the patient data in an electronic file for registration. During the registration process, interviews will be conducted to collect each patient’s baseline information, including physical examination findings, comprehensive medical history (with specific items, such as previous abdominal surgery and spinal disease), lifestyle habits, and medication history. These variables will be systematically included in the electronic registration form for all patients, allowing the assessment of whether such factors influenced the choice of shunt procedure (ie, VPS, LPS, or VAS) in subsequent analyses. The information mentioned subsequently will be recorded during the treatment and follow-up.

Physical and cognitive function assessments include mRS, 3-m Timed Up and Go Test, Mini-Mental State Examination, and Idiopathic Normal Pressure Hydrocephalus Grading Scale.

The subjective assessment includes questionnaire survey. The objective assessments include (1) CSF opening and closing pressure and its drainage volume during the lumber tap test, (2) general CSF laboratory data, (3) head computed tomography imaging, (4) head magnetic resonance imaging or magnetic resonance angiography, (5) shunt valve pressure settings, and (6) adverse events.

**Figure 2:**
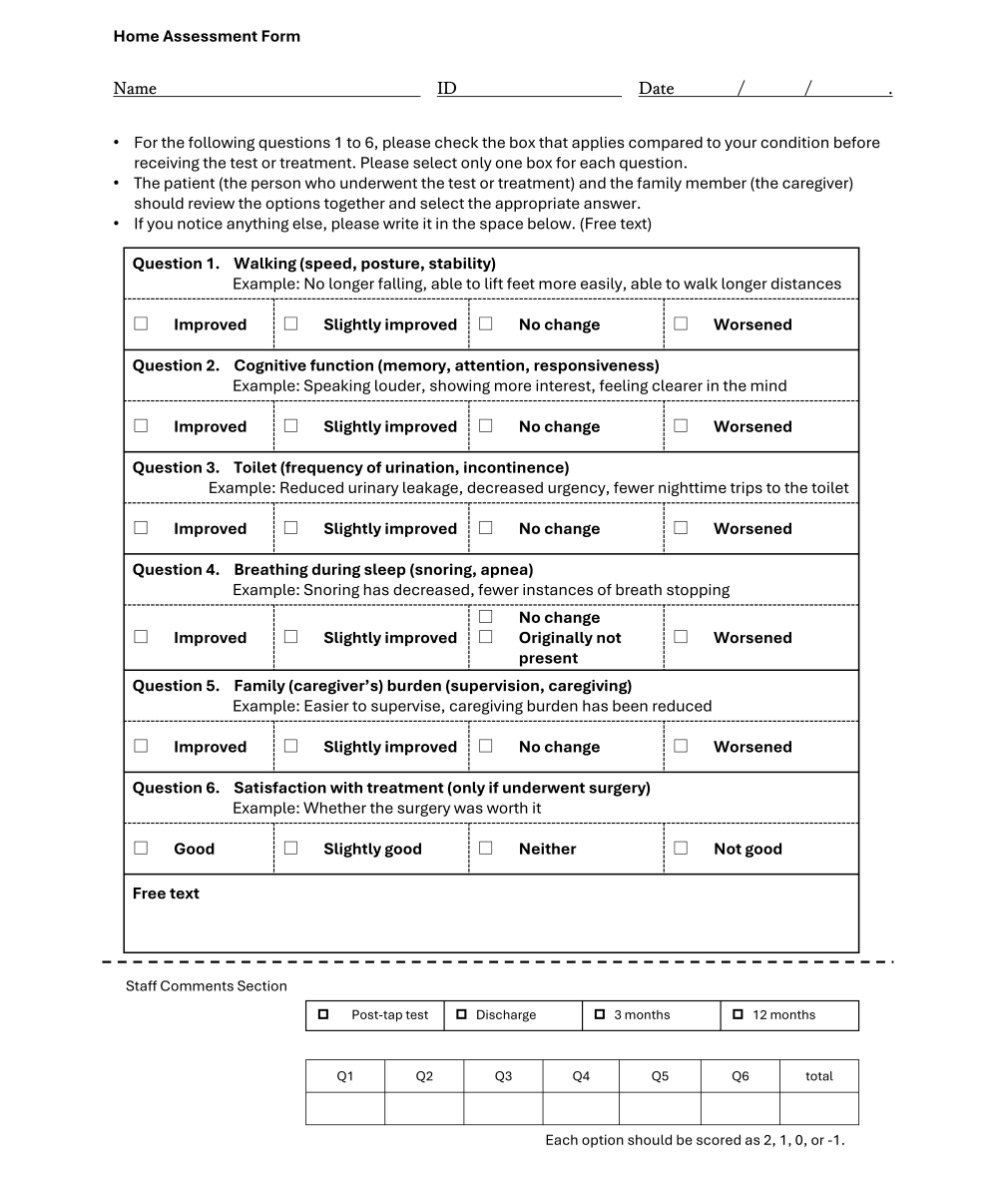
Patient home assessment form used in this multicenter prospective study of idiopathic normal pressure hydrocephalus. This questionnaire-based assessment tool, completed by patients and their families, enables the evaluation of the subjective changes after the tap test or shunt surgery. The form consists of 6 categories: gait, cognition, urination, snoring, caregiving, and overall satisfaction with the surgery.

The questionnaire survey that will be used in this study is an original tool for assessing patient-oriented outcomes. It consists of simple questionnaires regarding gait, cognitive and urinary functions, sleep, activities of daily living, and surgical satisfaction (Figure 2). The questionnaire uses a 4-choice ordinal scale to ensure that people with cognitive impairment can easily and reliably select the options. These forms will be completed as self-assessments by the patients and their family members.

### Surgical Decision and Procedures

The decision to perform shunt surgery is comprehensively determined based on the patient’s intention, condition, and the need for surgery. The reasons for the decision to undergo surgery will be documented. When shunt surgery is planned, the choice of procedure (ie, VPS, LPS, or VAS) will be determined based on the patient’s condition and the surgeon’s preference and technique. The type and configuration of the shunt system, including the choice of the catheter, valve, and valve pressure settings, will also be left to the discretion of each surgeon. However, the use of programmable valves equipped with antisiphon or antigravity devices is strongly recommended to minimize the risk of CSF overdrainage. Neither the manufacturer nor the model of the shunt system is specified in the study protocol. As CSF overdrainage after shunt surgery reportedly correlates with patient height, the height and weight of all patients will be recorded in the registry to allow for post hoc analysis of the relationship between body size and drainage volume. This study will provide no instructions or guidance regarding the surgical techniques or procedures.

### Imaging Analysis

Head computed tomography and magnetic resonance imaging scans will be collected as digital data. The analysis will be conducted under the guidance of the department of radiological sciences at Nagoya University Hospital. These imaging data will be quantitatively evaluated using dedicated image analysis software, including measurements of the DESH score, callosal angle, ventricular volume, and brain center-of-mass displacement. The impact of various factors, including patient background, shunt surgery effectiveness, and adverse events, will be analyzed using imaging findings.

### Representative Example of Clinical Protocol

**Figure 3:**
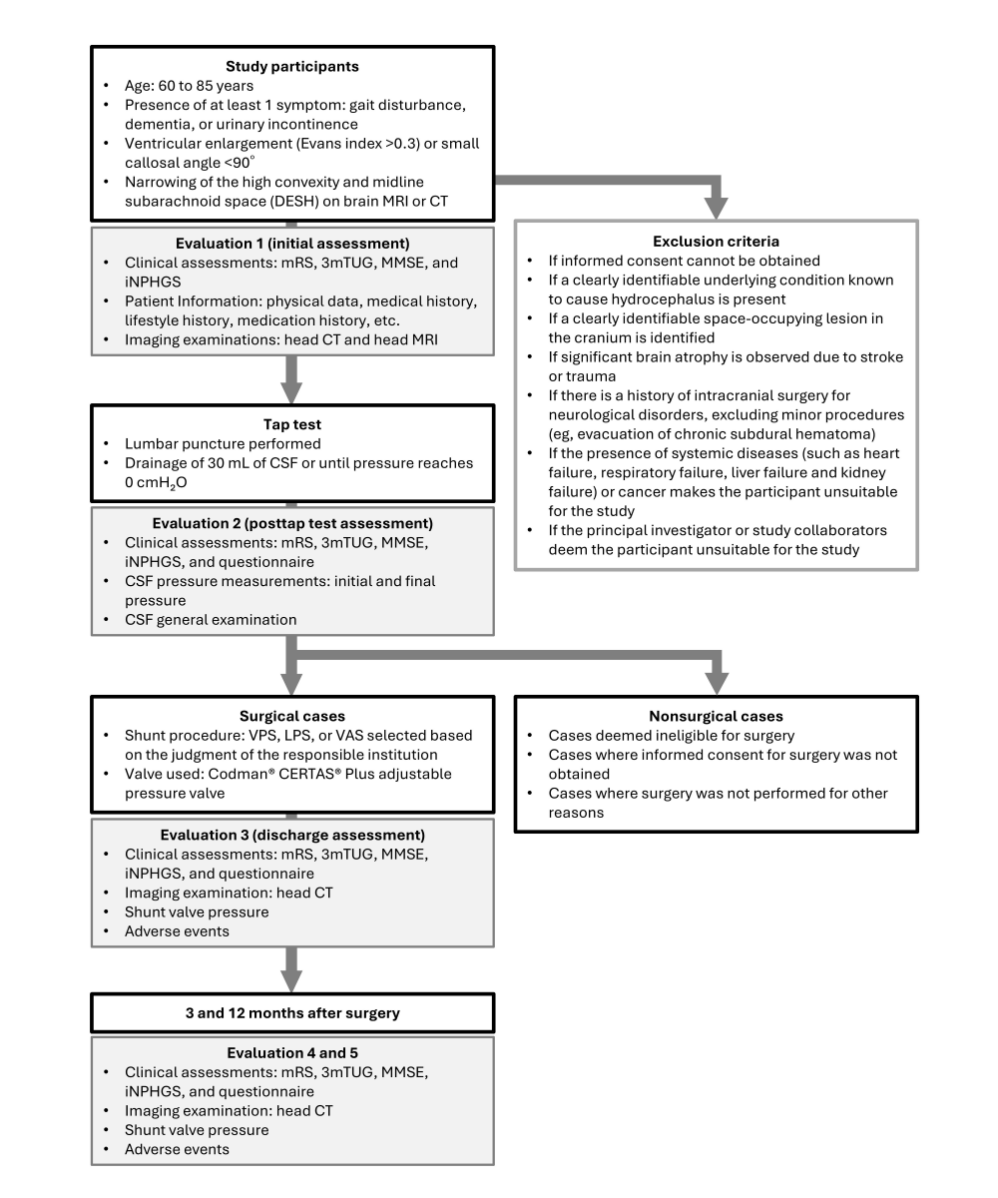
Flowchart illustrating the recommended protocol for diagnosis and assessment in this multicenter prospective study of idiopathic normal pressure hydrocephalus. The evaluation items for clinical symptoms include Modified Rankin Scale (mRS), 3-m Timed Up and Go (3mTUG) test, Mini-Mental State Examination (MMSE), Idiopathic Normal Pressure Hydrocephalus Grading Scale (iNPHGS), and subjective assessments via questionnaires completed by patients and their families. Head computed tomography (CT) imaging, shunt valve pressure settings, and adverse events are also collected and evaluated. The assessment is continued for 12 months after surgery. CSF: cerebrospinal fluid; DESH: disproportionately enlarged subarachnoid space hydrocephalus; LPS: lumboperitoneal shunt; MRI: magnetic resonance imaging; VAS: ventriculoatrial shunt; VPS: ventriculoperitoneal shunt.

As this is an observational study, there are no restrictions on the clinical guidelines or decisions. However, to avoid variability and confusion among participating institutions, clinical recommendations will be provided to each institution, and actual clinical practice may be conducted based on these recommendations. The recommended protocol was developed in accordance with the guidelines for the management of iNPH published by the Japanese Society of Normal Pressure Hydrocephalus. It incorporates the active use of the tap test. The guidelines recommend evaluating patients before and after the tap test, within 1 week, and at 3 and 12 months after shunt surgery (Figure 3).

### Study Registration

This study was registered in the Japan Registry of Clinical Trials, operated by Japan’s Ministry of Health, Labour and Welfare (jRCT1040250005).

### Ethical Considerations

Ethics approval was obtained from the ethical review board for life sciences of Nagoya University (2024-0286-2). The purpose of this study will be explained to the participants, and their participation will be entirely voluntary, with the right to withdraw from the study at any time. In cases where participants lack sufficient decision-making capacity, the information will be provided to a legally authorized representative. Informed consent will be obtained in writing from all participants before data collection. Participants will be assigned a study registration ID, and no personally identifiable information will be collected. All information will be stored in a locked cabinet to ensure confidentiality. Participants will not receive any compensation for their participation. Participants will be asked to provide consent to store their samples and data for future use. The results will be published in international peer-reviewed journals.

## Results

The patient enrollment for this study began in November 2024, following approval by the ethics committee. As of May 2025, approximately 60 participants have been enrolled from 11 institutions. Data collection is ongoing and is expected to be completed by December 2026. There will be a 1-year follow-up period. The main study results are anticipated to be published in 2028.

## Discussion

### Anticipated Findings

This protocol outlines a multicenter prospective observational study designed to comparatively evaluate the effectiveness and safety of 3 shunt procedures (ie, VPS, LPS, and VAS) for iNPH under real-world conditions. Building on the study objective stated in the Introduction section, the registry will standardize clinical assessments (eg, mRS, gait measures, and patient-oriented questionnaires) and incorporate structured imaging evaluations (eg, Evans index, callosal angle, and DESH-related features) while capturing the determinants of surgical decision-making (eg, previous abdominal or spinal surgery, body habitus, and surgeon experience). This design is intended to generate practice-informative comparative data to guide patient-tailored shunt selection.

### Interpretation and Comparison With Literature

iNPH has been increasingly recognized as a major neurological disorder in aging societies, alongside Alzheimer and Parkinson diseases, necessitating appropriate clinical management. Unlike other neurodegenerative diseases, no pharmacological treatment has been established for iNPH, making surgical intervention the only therapeutic option [17-22]. Therefore, careful selection of candidates for shunt surgery is essential. Understanding the characteristics of the 3 main shunt procedures is crucial for optimizing treatment outcomes.

Several studies have compared the outcomes of different shunt procedures for iNPH [16,23-27]. These studies generally reported no significant differences in therapeutic efficacy among the VPS, LPS, and VAS procedures. However, variations in complication rates have been observed across studies. Notably, some reports have suggested that VAS may be associated with a lower incidence of complications than VPS and LPS [24,26]. However, complications related to catheter placement in the venous system have also been reported, making the overall evaluation of VAS more challenging [28-30].

VPS is the first procedure that neurosurgeons typically learn and remains the most commonly performed technique among the 3 shunt types. Due to its widespread use, it is considered a reliable procedure for the management of various forms of hydrocephalus. LPS, particularly in Japan, is frequently selected for patients with iNPH, and the number of cases is as large as that for VPS procedures. In contrast, the VAS is less commonly used and is generally reserved for cases in which abdominal surgery is contraindicated or should be avoided. Recently, the cervical approach to VAS placement has become less invasive. The traditional technique of performing a wide-neck incision and cutting down the facial vein for catheter placement has been replaced by a more refined method involving percutaneous puncture and the use of peel-away introducers for internal jugular vein catheterization. This advancement has simplified the procedure, making it easier for neurosurgeons to master. Moreover, this technique is expected to reduce surgery-related complications, leading to a gradual increase in the number of cases for which VAS is actively selected.

This new information regarding VAS highlights the possibility of achieving therapeutic outcomes equivalent to those of VPS and LPS, potentially reducing complication rates. Such findings could position VAS as a first-line surgical treatment for iNPH. A comparative analysis of these 3 procedures may offer critical insights for developing guidelines to tailor surgical approaches based on individual patient backgrounds. To our knowledge, no previous studies have comprehensively compared these 3 procedures, making this investigation a novel endeavor.

### Limitations

This is a prospective observational study rather than a randomized controlled trial, making it difficult to compare the results across different surgical methods. One reason for this approach was to investigate the selection of surgical procedures for real-world clinical practice. Another reason was that randomization requires minimization of variability in baseline characteristics, resulting in reduced sample size and prolonged study period. Other limitations should be acknowledged. First, this study cannot completely exclude secondary causes of normal pressure hydrocephalus, such as stroke, meningitis, or collagen vascular diseases. Moreover, certain neurodegenerative disorders, including progressive supranuclear palsy and corticobasal degeneration, may coexist with or mimic iNPH, making clinical differentiation challenging [31,32]. Although the diagnostic criteria for iNPH will be strictly applied, the absence of pathological confirmation remains a limitation. Therefore, the influence of antecedent and comorbid diseases must be considered when interpreting imaging findings and clinical outcomes.

### Conclusions

By systematically comparing VPS, LPS, and VAS in a multicenter setting, this study is expected to clarify procedure-specific advantages and guide evidence-based surgical decision-making. Incorporating detailed clinical, imaging, and demographic factors may enable the identification of patient subgroups that benefit the most from each procedure. Furthermore, the findings could contribute to the refinement of future clinical guidelines and support the development of personalized treatment strategies for iNPH, ultimately improving long-term outcomes in this growing patient population.

## Data Availability

This paper presents a study protocol. The datasets generated or analyzed during this study not publicly available due to patient privacy and ethical restrictions but are available from the corresponding author on reasonable request.

## Abbreviations

CSF: cerebrospinal fluid
DESH: disproportionately enlarged subarachnoid space hydrocephalus
iNPH: idiopathic normal pressure hydrocephalus
LPS: lumboperitoneal shunt
mRS: Modified Rankin Scale
SINPHONI: Study of Idiopathic Normal Pressure Hydrocephalus on Neurological Improvement
VAS: ventriculoatrial shunt
VPS: ventriculoperitoneal shunt
